# Effects of COPD on in‐hospital outcomes of transcatheter aortic valve implantation: Results from the National Inpatient Sample database

**DOI:** 10.1002/clc.23475

**Published:** 2020-10-22

**Authors:** Fei Xiao, Jue Yang, Ruixin Fan

**Affiliations:** ^1^ Department of Cardiovascular Surgery Guangdong Provincial People's Hospital Guangdong China; ^2^ Guangdong Academy of Medical Sciences Guangdong China; ^3^ Guangdong Cardiovascular Institute Guangdong China; ^4^ Guangdong Provincial Key Laboratory of South Structural Heart Disease Guangdong China

**Keywords:** aortic valve stenosis, chronic obstructive pulmonary disease, National Inpatient Sample, postoperative complication, Transcatheter aortic valve implantation

## Abstract

**Background:**

Comorbid chronic obstructive pulmonary disease (COPD) increases morbidity and mortality among aortic valve replacement patients undergoing conventional surgery. The impact of COPD in patients undergoing less invasive transcatheter aortic valve insertion (TAVI) is unclear.

**Hypothesis:**

This study evaluates the in‐hospital outcomes of TAVI in patients with and without COPD.

**Methods:**

This population‐based, retrospective study of 8466 TAVI patients (29.87% with COPD) evaluates the effects of COPD on short‐term clinical outcomes (in‐hospital mortality, length of hospital stay, and postoperative complications) using data from the National Inpatient Sample database from 2011 to 2014. Logistic regression analysis was used to determine factors associated with in‐hospital mortality and postoperative complications. Linear regression analysis was used to identify factors associated with length of hospital stay.

**Results:**

COPD is significantly associated with increased risk of respiratory complications and pneumonia after TAVI (aOR = 1.43, 95% CI: 1.24‐1.64; *P* < .001) but not in‐hospital mortality, length of hospital stay, or non‐respiratory postoperative complications as compared to non‐COPD patients. Concomitant COPD is significantly associated with increased risk of respiratory complications or pneumonia after TAVI but may still be the best treatment option for some patients.

**Conclusions:**

Patients with comorbid COPD who receive TAVI have greater risk of developing postoperative respiratory complications and pneumonia. Vigilance for specific respiratory complications is highly warranted when treating this subgroup. Treatment decisions must be individualized.

## 1 INTRODUCTION

Chronic obstructive pulmonary disease (COPD) describes a constellation of conditions, including emphysema, chronic bronchitis, and refractory asthma, characterized by progressive airflow limitation and reduced gas exchange that is not fully reversible. In developed countries, the prevalence of COPD is high, estimated to be 8% to 10% among adults 40 years of age and older.^1^ COPD patients have a high burden of co‐morbid chronic illness, suggesting that overarching systemic processes underlie these conditions.^2^ Aortic stenosis (AS) is a common cardiovascular comorbidity among COPD patients, and respiratory complications may compromise postoperative morbidity and mortality in those who must undergo surgical treatment for AS.^3^ In AS, the increased LV pressure required to maintain cardiac output leads to ventricular hypertrophy, resulting in systolic and diastolic dysfunction,^4^ making valve replacement the only effective treatment.^5^

Comorbid COPD is associated with morbidity and mortality after open‐chest cardiac surgery.^6^, ^7^ Transcatheter aortic valve implantation (TAVI) became available as an alternative to conventional surgical aortic valve replacement (SAVR) in 2012 with the FDA approval of a device indicated for transfemoral or transapical delivery in patients with AS in whom open aortic valve replacement is precluded.^8^, ^9^ In a randomized, controlled trial confirming that clinical outcomes of TAVI are similar to those of SAVR,^10^ clinicians are advised to decide between these procedures, taking into account the effects of COPD on outcomes.

In pre‐surgical assessments of patients with severe aortic stenosis, the presence of COPD can contribute to a high preoperative risk score, potentially influencing the treatment choice toward TAVI rather than conventional surgery.^11^ However, the risk to COPD patients undergoing TAVI is still uncertain. Compared to conventional SAVR, TAVI results in significantly fewer respiratory complications in COPD patients because the duration of mechanical ventilation is shorter.^12^ However, another study showed that among patients with COPD, TAVI did not reduce the occurrence of postoperative pulmonary complications as compared to SAVR.^3^ Furthermore, several studies have shown that COPD is associated with higher mortality rates among TAVI patients.^11^, ^13^, ^14^ Given these conflicting results and serious implications regarding mortality, further investigation in a large patient cohort is warranted. Therefore, we conducted this study to determine the impact of COPD on short‐term outcomes (in‐hospital mortality, length of hospital stay, and postoperative complications) of patients receiving TAVI, using data from the National Inpatient Sample, the largest all‐payer US inpatient care database.

## 2 METHODS

This population‐based, retrospective observational study included hospitalized patients ≥18 years of age who underwent transcatheter aortic valve implantation (TAVI). Patient data were extracted from the HCUP‐NIS (2011‐2014) database using codes for the International Classification of Diseases, Ninth Revision (ICD‐9) (Table S1). The study cohort was further stratified according to the presence or absence of a diagnosis of chronic obstructive pulmonary disease (COPD).

The Nationwide Inpatient Sample (NIS) is the largest all‐payer U.S. inpatient care database that contains over one hundred clinical and nonclinical data elements from approximately 8 million hospital stays each year.^15^ Included in these data elements are primary and secondary diagnoses, primary and secondary procedures, admission and discharge status, patient demographics, expected payment source, length of stay, and hospital characteristics. All patients are considered for inclusion. The most recent NIS database contains data from about 1050 hospitals in 44 States in the U.S. sampled to approximate a 20% stratified sample of U.S. community hospitals as defined by the American Hospital Association. NIS was developed as part of the Healthcare Cost and Utilization Project (HCUP), which is sponsored by the Agency for Healthcare Research and Quality.

This study was approved by the institutional review board of Guangdong Provincial People's Hospital with certificate number HCUP‐29J72GZU8 and conforms to the data‐use agreement for the NIS from HCUP.^16^ (Overview of the Nationwide Inpatient Sample [NIS]. Available at: http://www.hcup-us.ahrq.gov/nisoverview.jsp) (Accessed December 2017).

The primary endpoints were in‐hospital mortality, respiratory complications and pneumonia, postoperative complications, and length of hospital stay. Respiratory complications and pneumonia and other postoperative complications defined by ICD‐9 codes and Clinical Classifications Software (CCS) codes^17^ are documented in Table S1.

Patient characteristics for covariate analysis included age, gender, race, income, insurance status, and transapical access (ICD‐9 procedure code 35.06). Comorbidities were identified from AHRQ comorbidity measures in the database determined by ICD‐9 diagnostic codes using algorithms validated by Elixhauser comorbidity scores.^18^ Hospital‐related characteristics (bed size, location/teaching status, and TAVI caseload) also were extracted from the NIS database.

Patient characteristics, hospital characteristics, comorbidities, and clinical outcomes are reported as n (weighted %) for categorical variables and mean ± SE (SE) for continuous variables according to COPD status. Comparative analysis was carried out using Pearson's Chi‐square test for categorical variables and the two‐sample *t*‐test for continuous variables. Univariate, simple logistic regression analysis was used to identify associations between outcomes (including clinical outcomes, in‐hospital mortality, postoperative complications, and respiratory complications or pneumonia) and COPD status, patient characteristics, hospital characteristics, and comorbidities. The results are presented as odds ratios (OR) with corresponding 95% confidence intervals (95% CI). Univariate simple linear regression analysis was used to identify associations between length of hospital stay and COPD status, patient characteristics, hospital characteristics, and comorbidities. Results are presented as *b*‐values with corresponding 95% CI. The results of multivariate model analysis are presented as adjusted OR (aOR) with 95% CI for clinical outcomes, in‐hospital mortality, postoperative complications, and respiratory complication/or pneumonia; and adjusted b value (adj. b) with 95% CI for model of length of hospital stay, separately. Weighted samples (DISCWT), stratum (NIS_STRATUM), and cluster (HOSPID) were used to generate national estimates for all analyses. All analyses were 2‐sided, and *P* < .05 was considered statistically significant. All statistical analyses were performed using the SAS software package version 9.4 (SAS Institute Inc., Cary, NC).

## 3 RESULTS

The final cohort included 8466 patients, 29.87% (2529/8466) of whom had COPD. Patient characteristics are shown in Table 1. Significant differences between patients with and without COPD were observed with respect to age, sex, income, insurance status, and transapical access (all *P* ≤ .036), and overall comorbidity. Specific comorbidities were significantly more prevalent among subjects with COPD, including depression, diabetes, fluid/electrolyte disorders, obesity, peripheral vascular disorders, and atrial fibrillation.

**TABLE 1:** Comparisons of baseline patient characteristics, comorbidities, and hospital characteristics between COPD and non‐COPD TAVI patients

	All patients (n = 8466)	COPD (n = 2529)	Non‐COPD (n = 5937)	*P* ^a^
*Patient characteristics*				
Age				**<.001**
<65 years	428 (5.1)	110 (4.4)	318 (5.4)	
65‐74 years	1114 (13.2)	454 (18)	660 (11.1)	
≥75 years	6924 (81.8)	1965 (77.7)	4959 (83.5)	
Sex				**<.001**
Female	4026 (47.6)	1124 (44.4)	2902 (48.9)	
Male	4440 (52.4)	1405 (55.6)	3035 (51.1)	
Race				.171
White	6868 (81.1)	2093 (82.8)	4775 (80.4)	
Non‐white	1009 (11.9)	270 (10.7)	739 (12.4)	
Unknown	589 (7)	166 (6.6)	423 (7.1)	
Income (percentile)				**.001**
0–25th	1729 (20.4)	578 (22.9)	1151 (19.4)	
26‐50th	2065 (24.4)	619 (24.5)	1446 (24.4)	
51–75th	2137 (25.3)	614 (24.3)	1523 (25.7)	
76–100th	2390 (28.2)	665 (26.3)	1725 (29)	
Unknown	145 (1.7)	53 (2.1)	92 (1.5)	
Insurance status				**.036**
Medicare/Medicaid	7709 (91.1)	2319 (91.7)	5390 (90.8)	
Private/HMO	593 (7)	153 (6.1)	440 (7.4)	
Self‐pay/no‐charge/other	151 (1.8)	55 (2.2)	96 (1.6)	
Unknown	13 (0.2)	2 (0.1)	11 (0.2)	
Transapical access				**<.001**
Yes	1713 (20.2)	585 (23.1)	1128 (19)	
No	6753 (79.8)	1944 (76.9)	4809 (81)	
*Hospital characteristics*				
Hospital bed size				.089
Small	399 (4.7)	100 (4)	299 (5)	
Medium	1356 (16.1)	413 (16.4)	943 (15.9)	
Large	6711 (79.2)	2016 (79.6)	4695 (79)	
Hospital location and status				**.002**
Rural	62 (0.7)	11 (0.4)	51 (0.9)	
Urban non‐teaching	865 (10.2)	235 (9.3)	630 (10.6)	
Urban teaching	7539 (89.1)	2283 (90.3)	5256 (88.5)	
Hospital annual TAVI caseload (percentile)				.347
0–25th	1816 (21.5)	532 (21)	1284 (21.6)	
26–50th percentile	2399 (28.4)	684 (27.1)	1715 (28.9)	
51–75th percentile	2128 (25.1)	660 (26.1)	1468 (24.7)	
76–100th percentile	2123 (25)	653 (25.8)	1470 (24.7)	
*Comorbidities*				
Anemia	2242 (26.5)	699 (27.6)	1543 (26)	.112
Collagen‐vascular diseases	412 (4.9)	138 (5.5)	274 (4.6)	.090
Congestive heart failure	999 (11.8)	312 (12.4)	687 (11.6)	.279
Coagulopathy	2018 (23.8)	556 (22)	1462 (24.6)	**.008**
Depression	608 (7.2)	219 (8.7)	389 (6.5)	**.001**
Diabetes	2919 (34.5)	942 (37.3)	1977 (33.3)	**<.001**
Hypertension	6737 (79.6)	2011 (79.5)	4726 (79.6)	.934
Hypothyroidism	1718 (20.3)	507 (20)	1211 (20.4)	.692
Liver disease	213 (2.5)	57 (2.2)	156 (2.6)	.287
Fluid/electrolyte disorders	2263 (26.7)	748 (29.6)	1515 (25.5)	**<.001**
Other neurological disorders	534 (6.3)	153 (6.1)	381 (6.4)	.507
Obesity	1188 (14)	483 (19.1)	705 (11.9)	**<.001**
Paralysis	149 (1.8)	44 (1.7)	105 (1.8)	.932
Peripheral vascular disorders	2504 (29.6)	904 (35.7)	1600 (27)	**<.001**
Pulmonary circulation disorders	315 (3.7)	105 (4.2)	210 (3.5)	.132
Renal failure	3062 (36.1)	941 (37.2)	2121 (35.7)	.203
Weight loss	435 (5.1)	129 (5.1)	306 (5.2)	.925
Atrial fibrillation	3786 (44.7)	1209 (47.7)	2577 (43.4)	**<.001**
Elixhauser comorbidity score (percentile)				**<.001**
0‐25th	794 (9.4)	45 (1.8)	749 (12.6)	
26‐50th	3137 (37.1)	636 (25.2)	2501 (42.1)	
51‐75th	1779 (21)	572 (22.6)	1207 (20.3)	
76–100th	2756 (32.6)	1276 (50.5)	1480 (24.9)	

*Note*: Categorical variables are presented as unweighted counts (weighted percentage). Bold values indicate a significant difference (*P* < .05).

^a^ COPD vs non‐COPD, according to Chi‐square analysis of categorical variables.

Clinical outcomes after TAVI (in‐hospital mortality, length of hospital stay, and postoperative complications) are compared between COPD and non‐COPD patients and are presented in Table 2. The in‐hospital mortality rate (overall, 4.2%) and length of hospital (overall mean, 8.06 days [SD, 0.12]) were similar between COPD and non‐COPD patients (both *P* > .05). The presence of (non‐respiratory) postoperative complications, respiratory complications or pneumonia (20.9% vs 15.5%), infection or sepsis (2.2% vs 3%), or device complications (2.1% vs 3%) differed between COPD and non‐COPD patients (all *P* ≤ .029) (Table 2).

**TABLE 2:** Comparison of clinical outcomes between COPD and non‐COPD TAVI patients

Clinical outcomes	All patients (n = 8466)	COPD (n = 2529)	Non‐COPD (n = 5937)	*P* ^a^
In‐hospital mortality	354 (4.2)	106 (4.2)	248 (4.2)	.982
Length of in‐hospital stay	8.06 ± 0.12	8.26 ± 0.17	7.98 ± 0.13	.092
Respiratory complications/pneumonia	1450 (17.1)	527 (20.9)	923 (15.5)	**<.001**
Non‐respiratory postoperative complication	4964 (58.6)	1519 (60.1)	3445 (58)	.078
Cardiovascular complications	1241 (14.7)	370 (14.6)	871 (14.7)	.942
Bleeding complications	3667 (43.3)	1124 (44.4)	2543 (42.8)	.168
Acute renal failure	1756 (20.7)	535 (21.2)	1221 (20.6)	.520
Infection/sepsis	231 (2.7)	55 (2.2)	176 (3)	**.029**
DVT/Pulmonary embolism	294 (3.5)	83 (3.3)	211 (3.5)	.529
Wound complications	408 (4.8)	117 (4.6)	291 (4.9)	.605
Device complications	232 (2.7)	53 (2.1)	179 (3)	**.016**
Need for ECMO	29 (0.3)	11 (0.4)	18 (0.3)	.400
Other complications	307 (3.6)	92 (3.6)	215 (3.6)	.990

*Note*: Data for continuous variables are presented as the mean ± SE. Data for categorical variables are presented as unweighted counts (weighted percentage). Bold values indicate significantly (*P* < .05).

^a^ As determined by Chi‐square test for categorical variables and t‐test for continuous variables.

### 3.1 In‐hospital mortality

Results of multivariate analysis showed that female sex, income level at the 51st to 75th percentile (vs 76th to 100th percentile), treatment in a hospital at the lowest percentile of annual TAVI caseload, coagulopathy, fluid/electrolyte disorders, paralysis, renal failure, and weight loss were associated with increased in‐hospital mortality. Conversely, anemia, depression, and hypertension were associated with lower odds of patient in‐hospital mortality (all *P* < .05) (Table 3).

**TABLE 3:** The effect of patients' COPD status, comorbidities and other characteristics on mortality and length of stay

Variables	In‐hospital mortality		Length of hospital stay	
	OR (95% CI)	aOR (95% CI)	b (95% CI)	Adj. b (95% CI)
COPD (yes vs no)	1.00 (0.80‐1.26)	1.03 (0.79‐1.33)	0.29 (−0.05 to 0.62)	−0.11 (−0.47 to 0.24)
*Patient characteristics*				
Age				
<65 years	Ref.	Ref.	0	0
64‐74 years	0.97 (0.57‐1.65)	1.17 (0.63‐2.16)	−1.15 (−2.25 to 0.06)*	−1.09 (−2.19 to 0.02)
≥75 years	1.07 (0.69‐1.68)	1.17 (0.66‐2.05)	−1.38 (−2.39 to 0.36)**	−1.63 (−2.72 to 0.53)**
Sex				
Male	Ref.	Ref.	0	0
Female	1.37 (1.1‐1.7)**	1.52 (1.2‐1.92)**	0.61 (0.3‐0.92)***	0.57 (0.28‐0.86)***
Race				
Non‐white	Ref.	Ref.	0	0
White	0.94 (0.68‐1.31)	1 (0.71‐1.4)	−1.39 (−2.01 to 0.78)***	−0.93 (−1.47 to 0.38)**
Income				
76‐100th percentile	Ref.	Ref.	0	0
51‐75th percentile	0.74 (0.55‐0.98)*	0.72 (0.53‐0.97)*	−0.33 (−0.9 to 0.23)	−0.22 (−0.7 to 0.26)
26‐50th percentile	0.72 (0.55‐0.96)*	0.76 (0.57‐1.02)	−1.09 (−1.61 to 0.57)***	−0.78 (−1.25 to 0.32)**
0‐25th percentile	0.79 (0.58‐1.07)	0.8 (0.57‐1.11)	−0.51 (−1.09 to 0.07)	−0.48 (−1 to 0.05)
Insurance status				
Medicare/Medicaid	Ref.	Ref.	0	0
Private/HMO	0.97 (0.64‐1.46)	1.11 (0.67‐1.82)	−0.39 (−1.02 to 0.25)	−0.51 (−1.19 to 0.16)
Self‐pay/no‐charge/other	1.11 (0.49‐2.55)	1.33 (0.55‐3.2)	0.49 (−0.83 to 1.82)	0.43 (−0.68 to 1.55)
Transapical access	1.41 (1.08‐1.82)*	1.13 (0.85‐1.5)	2.4 (1.97‐2.84)***	1.67 (1.29‐2.06)***
*Hospital characteristics*				
Hospital bed size				
Large	Ref.	Ref.	0	0
Small	1.17 (0.67‐2.05)	0.98 (0.55‐1.74)	−0.93 (−1.7−−0.17)*	−0.91(−1.58‐0.25)**
Medium	1.08 (0.81‐1.45)	1.02 (0.75‐1.38)	−0.31 (−0.86‐0.24)	−0.23 (−0.72‐0.27)
Hospital location and status				
Urban teaching	Ref.	Ref.	0	0
Rural	0.77 (0.29‐2.05)	0.92 (0.35‐2.47)	−2.42 (−3.05−−1.79)***	−1.51 (−2.36−−0.66)***
Urban non‐teaching	1.09 (0.8‐1.48)	0.97 (0.71‐1.34)	−0.94 (−1.68−−0.2)*	−0.77 (−1.44−−0.11)*
Hospital annual TAVI caseload				
76‐100th percentile	Ref.	Ref.	0	0
51‐75th percentile	1.06 (0.76‐1.47)	1.01 (0.71‐1.42)	0.52 (−0.28‐1.32)	0.33 (−0.35‐1.01)
26‐50th percentile	1.39 (1.05‐1.86)*	1.35 (0.99‐1.84)	0.28 (−0.38‐0.94)	0.26 (−0.3‐0.81)
0‐25th percentile	1.4 (1.04‐1.89)*	1.44 (1.04‐1.99)*	−0.2 (−0.85‐0.46)	−0.2 (−0.77‐0.37)
*Comorbidities*				
Anemia	0.79 (0.61‐1.02)	0.68 (0.5‐0.92)*	0.94 (0.58‐1.31)***	0.12 (−0.29‐0.53)
Collagen‐vascular diseases	0.62 (0.34‐1.13)	0.58 (0.31‐1.1)	−0.5 (−1.1‐0.09)	−0.72 (−1.32−−0.13)*
Congestive heart failure	1.59 (1.19‐2.12)**	1.26 (0.84‐1.88)	1.62 (0.95‐2.29)***	0.73 (0.09‐1.38)*
Coagulopathy	1.93 (1.53‐2.44)***	1.68 (1.28‐2.2)***	1.77 (1.34‐2.2)***	0.8 (0.35‐1.24)***
Depression	0.48 (0.28‐0.84)*	0.53 (0.3‐0.95)*	0.14 (−0.41‐0.69)	0.04 (−0.52‐0.6)
Diabetes	0.67 (0.52‐0.86)**	0.77 (0.57‐1.03)	−0.24 (−0.55‐0.07)	−0.16 (−0.53‐0.21)
Hypertension	0.41 (0.33‐0.52)***	0.47 (0.36‐0.61)***	−2.3 (−2.81−−1.79)***	−2.3 (−2.78−−1.83)***
Hypothyroidism	0.9 (0.69‐1.16)	0.92 (0.67‐1.25)	−0.24 (−0.63‐0.15)	−0.43 (−0.86‐0.01)
Liver disease	1.88 (1.13‐3.12)*	1.7 (0.97‐2.97)	1.05 (−0.1‐2.2)	−0.07 (−1.08‐0.94)
Fluid/electrolyte disorders	2.82 (2.26‐3.52)***	2.27 (1.73‐2.98)***	4.94 (4.46‐5.42)***	3.64 (3.14‐4.14)***
Other neurological disorders	1.09 (0.68‐1.74)	1 (0.6‐1.66)	0.63 (0.02‐1.23)*	0.37 (−0.23‐0.96)
Obesity	0.66 (0.47‐0.93)*	0.78 (0.53‐1.13)	−0.3 (−0.75‐0.16)	−0.26 (−0.74‐0.22)
Paralysis	2.87 (1.71‐4.79)***	2.19 (1.22‐3.92)**	4.22 (2.53‐5.9)***	2.89 (1.39‐4.39)***
Peripheral vascular disorders	1.17 (0.93‐1.47)	1.2 (0.91‐1.59)	0.32 (−0.03‐0.66)	−0.07 (−0.42‐0.27)
Pulmonary circulation disorders	2.24 (1.48‐3.38)***	1.62 (0.96‐2.72)	3.15 (1.7‐4.6)***	1.63 (0.27‐2.99)*
Renal failure	1.41 (1.14‐1.74)**	1.57 (1.21‐2.03)**	1.78 (1.43‐2.13)***	1.48 (1.09‐1.88)***
Weight loss	4.02 (2.96‐5.46)***	2.42 (1.69‐3.45)***	9.07 (7.86‐10.29)***	7 (5.88‐8.12)***
Atrial fibrillation	1.11 (0.9‐1.37)	1 (0.8‐1.25)	1.39 (1.08‐1.7)***	1.06 (0.79‐1.32)***
Elixhauser comorbidity score				
76‐100th percentile	Ref.	Ref.	0	0
51‐75th percentile	0.75 (0.57‐0.98)*	0.96 (0.67‐1.39)	−1.62 (−2.08−−1.17)***	−0.24 (−0.79‐0.32)
26‐50th percentile	0.6 (0.47‐0.77)***	0.94 (0.57‐1.54)	−2.69 (−3.07−−2.3)***	−0.34 (−1.12‐0.44)
0‐25th percentile	0.88 (0.61‐1.26)	1.37 (0.66‐2.84)	−3.32 (−3.88−−2.76)***	−0.93 (−2.13‐0.26)

*Note*: Multivariate models were adjusted for patient characteristics, hospital characteristics and comorbidities. The multivariate analysis might be limited with the lower collinearity of Elixhauser comorbidity score.

* *P* < .05.

** *P* < .01.

*** *P* < .001.

### 3.2 Length of stay

Results of multivariate analysis showed that shorter hospital stays correlated with patient age ≥ 75 years, white race, income level at the 26th to 50th percentile (vs the highest percentile), treatment at hospitals with small bedsize, rural or urban non‐teaching hospital, collagen‐vascular disease, and hypertension. Conversely, longer hospital stays correlated with female sex, insurance status of self‐pay/no‐charge/other, congestive heart failure, coagulopathy, fluid/electrolyte disorders, paralysis, renal failure, weight loss, and atrial fibrillation (all *P* < .05) (Table 3).

### 3.3 Non‐respiratory postoperative complications

Results of multivariate analysis showed increased odds of non‐respiratory postoperative complications associated with female sex, transapical access in TAVI, treatment in a hospital at the lowest percentile of annual TAVI caseload, and comorbidities, including coagulopathy, fluid/electrolyte disorders, paralysis, peripheral vascular disorders, renal failure, and weight loss. Hypertension and obesity were associated with lower odds of postoperative complications (all *P* < .05) (Table 4).

**TABLE 4:** The effect of patients' COPD status, comorbidities and other characteristics on respiratory and overall postoperative complications

Variables	Non‐respiratory postoperative complications		Respiratory complications/pneumonia	
	OR (95% CI)	aOR (95% CI)	OR (95% CI)	aOR (95% CI)
COPD (yes vs no)	1.09 (0.99, 1.2)	1.05(0.94‐1.18)	1.43(1.27‐1.61)***	1.43 (1.24–1.64)***
*Patient characteristics*				
Age				
<65 years	Ref.	Ref.	Ref.	Ref.
64–74 years	0.95 (0.76‐1.18)	1.01 (0.78‐1.3)	0.93 (0.72‐1.21)	0.97 (0.72‐1.33)
≥75 years	1.13 (0.94‐1.37)	1.15 (0.9‐1.46)	0.77 (0.61‐0.96)*	0.77 (0.58‐1.02)
Sex				
Male	Ref.	Ref.	Ref.	Ref.
Female	1.3 (1.19‐1.41)***	1.42 (1.3‐1.57)***	1.26 (1.13‐1.41)***	1.32 (1.17‐1.48)***
Race				
Non‐white	Ref.	Ref.	Ref.	Ref.
White	0.77 (0.65‐0.9)**	0.86 (0.72‐1.01)	0.83 (0.69‐1)	0.66 (0.46‐0.95)*
Income				
76‐100th percentile	Ref.	Ref.	Ref.	Ref.
51‐75th percentile	0.91 (0.79‐1.05)	0.9 (0.78‐1.05)	1.01 (0.86‐1.18)	0.99 (0.84‐1.17)
26‐50th percentile	0.89 (0.77‐1.02)	0.9 (0.78–1.05)	0.98 (0.83‐1.15)	1.01 (0.85‐1.2)
0‐25th percentile	1.01 (0.86–1.18)	1 (0.85‐1.18)	1.08 (0.91‐1.27)	1.06 (0.88‐1.28)
Insurance status				
Medicare/Medicaid	Ref.	Ref.	Ref.	Ref.
Private/HMO	0.93 (0.77‐1.13)	1.08 (0.86‐1.37)	1.07 (0.87‐1.33)	1.04 (0.81‐1.33)
Self‐pay/no‐charge/other	1.16 (0.78‐1.72)	1.3 (0.89‐1.89)	1.16 (0.71‐1.91)	1.15 (0.69‐1.92)
Transapical access	1.82 (1.6‐2.06)***	1.56 (1.37‐1.78)***	2 (1.74‐2.3)***	1.65 (1.42‐1.91)***
*Hospital characteristics*				
Hospital bed size				
Large	Ref.	Ref.	Ref.	Ref.
Small	1.11 (0.81‐1.52)	1.06 (0.82‐1.36)	0.76 (0.5‐1.15)	0.65 (0.43‐0.99)*
Medium	1.25(1.05‐1.5)*	1.19(0.99‐1.43)	1.08(0.91‐1.29)	1 (0.83‐1.2)
Hospital location and status				
Urban teaching	Ref.	Ref.	Ref.	Ref.
Rural	0.8 (0.56‐1.14)	0.89(0.62‐1.29)	0.51(0.35‐0.75)**	0.52 (0.32‐0.83)**
Urban non‐teaching	0.97(0.83‐1.13)	0.91(0.78‐1.06)	0.93(0.75‐1.15)	0.81 (0.65‐1.01)
Hospital annual TAVI caseload				
76–100th percentile	Ref.	Ref.	Ref.	Ref.
51–75th percentile	1.18(0.95‐1.47)	1.07 (0.87‐1.33)	1.42 (1.14‐1.76)**	1.37 (1.09‐1.73)**
26–50th percentile	1.23 (1.03‐1.48)*	1.19 (0.99‐1.42)	1.68 (1.37‐2.07)***	1.75 (1.39‐2.2)***
0–25th percentile	1.35 (1.12‐1.62)**	1.28 (1.06‐1.53)**	1.81 (1.49‐2.19)***	1.86 (1.49‐2.32)***
*Comorbidities*				
Anemia	1.21 (1.08‐1.36)**	0.91 (0.79–1.05)	1.05 (0.93‐1.2)	0.88 (0.75‐1.03)
Collagen‐vascular diseases	1.14 (0.92‐1.4)	1.13 (0.91‐1.4)	0.96 (0.74‐1.24)	0.9 (0.68‐1.2)
Congestive heart failure	1.03 (0.9‐1.17)	0.98 (0.84‐1.16)	1.32 (1.11‐1.56)**	1.25 (1.01‐1.55)*
Coagulopathy	2.34 (2.09‐2.62)***	2.08 (1.82‐2.37)***	2.18 (1.92‐2.48)***	1.97 (1.69‐2.29)***
Depression	1.05 (0.89‐1.23)	1.02 (0.85‐1.22)	1.06 (0.86–1.3)	1.01 (0.8‐1.27)
Diabetes	0.92 (0.84–1)	0.93 (0.83‐1.05)	0.88 (0.78‐0.99)*	0.93 (0.81‐1.07)
Hypertension	0.82 (0.74‐0.91)***	0.72 (0.63‐0.83)***	0.56 (0.49‐0.64)***	0.56 (0.47‐0.65)***
Hypothyroidism	1.02 (0.92‐1.13)	0.95 (0.83‐1.08)	0.95 (0.83‐1.09)	0.95 (0.81‐1.12)
Liver disease	1.13 (0.85–1.5)	0.92 (0.67‐1.26)	1.26 (0.87‐1.83)	0.93 (0.62‐1.4)
Fluid/electrolyte disorders	3.21 (2.87‐3.6)***	2.61 (2.3‐2.96)***	3.22 (2.83‐3.66)***	2.57 (2.19‐3)***
Other neurological disorders	1.04 (0.89‐1.22)	0.97 (0.8‐1.17)	1.01 (0.81‐1.27)	0.94 (0.73‐1.2)
Obesity	0.81 (0.71‐0.92)**	0.81 (0.7‐0.94)**	0.86 (0.73‐1.02)	0.84 (0.7‐1.02)
Paralysis	2.42 (1.65‐3.55)***	2.2 (1.45‐3.35)***	2.14 (1.48‐3.08)***	1.78 (1.17‐2.72)**
Peripheral vascular disorders	1.34 (1.21‐1.48)***	1.24 (1.1‐1.4)***	1.19 (1.04‐1.37)*	1.13 (0.96‐1.33)
Pulmonary circulation disorders	1.11 (0.88‐1.39)	0.96 (0.73‐1.25)	1.54 (1.19–2)**	1.17 (0.86‐1.61)
Renal failure	1.99 (1.79‐2.2)***	2.02 (1.78‐2.3)***	1.22 (1.08‐1.38)**	1.24 (1.06‐1.44)**
Weight loss	3.35 (2.52‐4.44)***	2.24 (1.65‐3.06)***	4.76 (3.91‐5.8)***	3.42 (2.71‐4.32)***
Atrial fibrillation	1.18 (1.09‐1.28)***	1.08 (0.98‐1.18)	1.13 (1.01‐1.26)*	1.02 (0.9‐1.15)
Elixhauser comorbidity score				
76‐100th percentile	Ref.	Ref.	Ref.	Ref.
51‐75th percentile	0.66 (0.58‐0.75)***	0.92 (0.78‐1.1)	0.68 (0.59‐0.8)***	1 (0.81‐1.23)
26‐50th percentile	0.47 (0.42‐0.53)***	0.9 (0.72‐1.13)	0.53 (0.47‐0.61)***	1.02 (0.77‐1.36)
0‐25th percentile	0.4 (0.34‐0.48)***	0.94 (0.66‐1.34)	0.44 (0.34‐0.57)***	0.95 (0.61‐1.46)

*Note*: Multivariate models were adjusted for patient characteristics, hospital characteristics and comorbidities. The multivariate analysis might be limited with the lower collinearity of Elixhauser comorbidity score.

* *P* < .05.

** *P* < .01.

*** *P* < .001.

### 3.4 Respiratory complications/pneumonia

Results of multivariate analysis showed that COPD, female sex, transapical access, hospital annual TAVI caseload (0‐25th, 26‐50th, and 51‐75th percentiles vs 76‐100th percentile), and comorbidities, including congestive heart failure, coagulopathy, fluid/electrolyte disorders, paralysis, renal failure, and weight loss (all *P* < .05), were significantly associated with increased odds of respiratory complications or pneumonia; in contrast, white race, small bedsize, rural hospital (vs urban teaching hospital), and hypertension were associated with lower odds of respiratory complications or pneumonia (all *P* < .05) (Table 4).

## 4 DISCUSSION

The present study evaluated the clinical characteristics and in‐hospital outcomes of patients undergoing TAVI with and without COPD. Statistical analysis of data for this cohort of 8466 patients (29.87% with COPD) revealed that COPD is significantly associated with increased risk of respiratory complications and pneumonia after TAVI. However, no significant differences were observed regarding in‐hospital mortality, length of hospital stay, or non‐respiratory postoperative complications between COPD and non‐COPD patients.

COPD is a common comorbidity among patients with AS and is a major predictor of adverse outcomes and greater mortality in patients undergoing open cardiac surgeries.^6^ In one study, 28% to 43% of patients undergoing TAVI were reported to have COPD.^14^ However, while Ando et al reported that respiratory‐related complications in patients with COPD were significantly fewer after TAVI than after SAVR,^12^ results of the present study showed that the risk of such complications is still significantly higher among these patients than among those without COPD. This comparison is particularly valid because the data analyzed in both studies were from the same NIS database, covering the same years. Comparison of these two studies also sheds light on the broader picture of in‐hospital mortality and non‐respiratory complications, revealing no differences between TAVI patients with and without COPD but significantly better outcomes for patients with COPD undergoing TAVI than those undergoing SAVR. In fact, Ando et al^12^ concluded that TAVR may be the preferable mode of aortic valve replacement in COPD patients.

Results of the present study show that the length of hospital stay and rates of non‐respiratory complications after TAVI are not significantly different between patients with and without COPD in the NIS cohort, consistent with the results of Ando et al. mentioned above.^12^ Patients without COPD also had shorter hospital stays after TAVI than after SAVR.^19^ Together, the findings of these two studies and ours suggest that COPD patients who undergo TAVI are at no greater risk of poor in‐hospital outcomes than those undergoing SAVR.

Outcomes of COPD patients also may be improved with the use of minimalist TAVR, which requires only minimal sedation instead of general anesthesia. Although the NIS did not include data of anesthesia to include in our analyses, minimalist TAVR is shown to result in less resource utilization and increased one‐year survival in patients with high surgical risk associated with severe COPD,^20^ suggesting that it is an especially appropriate option. Caixeta and Lemos^21^ also reported that using a minimalist approach to TAVI resulted in shorter hospital stays and improved short‐ and long‐term survival in selected COPD patients compared to the standard approach.

Studies that stratify patients by COPD severity, an option not possible using NIS data, report a higher risk of pulmonary complications and mortality after TAVI in patients with more severe COPD.^14^ This finding may help to explain some of the differences in mortality and in‐hospital outcomes reported between studies, as COPD cohorts may differ with respect to severity profile. For example, while one study reported no differences in 30‐day mortality between TAVI patients with and without COPD,^19^ other studies have reported higher post‐TAVI mortality in patients with COPD than in those without COPD.^11^, ^13^ This may be associated with higher baseline risk profiles of TAVI patients that may mask the benefits portended by TAVI.^3^ Thus, the complexity of COPD appears to confound direct comparison between study cohorts and underscores the importance of choosing treatments carefully on a case‐by‐case basis in this patient population.

An important strength of the present study is that the cohort includes the largest number of patients is such a study, selected from all geographical regions in the US and covering hospital admissions over a 4‐year period. In addition, we analyzed non‐respiratory and respiratory complications separately, an important distinction for COPD patients that has been determined less frequently in previous studies. This study also has several limitations. COPD and other comorbidities were defined based on the ICD‐9 coding system, which does not include the severity of COPD and other comorbidities. Also, the NIS database 2011‐2014 used for this study does not include current‐generation TAVI valves,^22^ which have improved over time. In addition, potential confounding variables such as types of anesthesia patients received and lifestyle factors were not collected by HCUP‐NIS and therefore could not be included in the analyses of the present study. This study focused mainly on in‐hospital outcomes. Late morbidity after discharge was not evaluated due to the nature of the database. Further well‐designed studies that include analysis of COPD severity and late morbidities are highly warranted.

Concomitant COPD in TAVI patients is significantly associated with increased risk of respiratory complications and pneumonia but may still be the best treatment option for some patients. Nevertheless, findings of the present study suggest that vigilance for respiratory complications and pneumonia during postoperative care is still highly warranted while managing this subgroup of patients. Treatment decisions regarding aortic valve replacement for COPD patients are not straightforward and must be made with careful consideration of each patient.

## CONFLICT OF INTEREST

The authors declare no potential conflict of interest.

## DATA AVAILABILITY

The datasets generated during the current study are available from the corresponding author on reasonable request.

## Supporting information


**Table S1**: Summary of the ICD codes used for data extraction.
