# Supplementary material for: Effects of COPD on in‐hospital outcomes of transcatheter aortic valve implantation: Results from the National Inpatient Sample database
Source: Clin Cardiol. 2020 Oct 22;43(12):1524–33. doi: 10.1002/clc.23475 (PMC7724217; doi:10.1002/clc.23475)
Supplement: Supplementary file 1 — Table S1: Summary of the ICD codes used for data extraction. [file CLC-43-1524-s001.docx]

Table S1 Summary of the ICD codes used for data extraction.

| **Item** | **ICD code** |
| --- | --- |
| **Respiratory complications & pneumonia** |  |
| Pulmonary insufficiency following trauma and surgery | 518.5 |
| Acute respiratory failure | 518.81 |
| Respiratory complications not elsewhere classified | 997.3 |
| Pneumonia | 122CSS |
| **Postoperative complication** |  |
| Cardiovascular complications | 997.1, 997.02, 997.09, 997.2, 997.7, 998.0, 100CCS |
| Bleeding complications | 285.1, 998.1, procedure code 99.0 |
| Acute renal failure | 584, 157CCS, V45.1 |
| Infection/sepsis | 998.5, 995.9 |
| DVT/PE | 451.11, 451.19, 451.2, 451.81-84, 451.89, 451.9, 453.40-42, 453.8, 453.9, 997.2 |
| Wound complications | 998.12-998.13, 998.3, 998.5 |
| Device complications | 996.1, 996.62, 996.74, 998.4, 998.7, 998.2 |
| Need for ECMO | procedure code 39.65 |
| Others (Digestive system complications, urinary complications, nervous system complications, persistent postoperative fistula, other specified complications of procedures not elsewhere classified, and unspecified complication of procedure) | 997.0, 997.4-997.5, 997.9, 998.6, 998.8-998.9 |
| **Patients' characteristics** |  |
| Chronic obstructive pulmonary disease | 491, 492, 493.2, 496 |
| Transcatheter aortic valve implantation | procedure code 35.05, 35.06 |
| Transapical access | procedure code 35.06 |
